# Understanding the relationship between egg- and antigen-based diagnostics of *Schistosoma mansoni* infection pre- and post-treatment in Uganda

**DOI:** 10.1186/s13071-017-2580-z

**Published:** 2018-01-08

**Authors:** Joaquín M. Prada, Panayiota Touloupou, Moses Adriko, Edridah M. Tukahebwa, Poppy H. L. Lamberton, T. Déirdre Hollingsworth

**Affiliations:** 1 Department of Mathematics, University of Warwick, Coventry, UK; 2 School of Veterinary Medicine, Faculty of Health and Medical Sciences, University of Surrey, Guildford, UK; 3 Department of Statistics, University of Warwick, Coventry, UK; 4 Vector Control Division, Ministry of Health, Uganda, Kampala, Uganda; 5 Institute of Biodiversity, Animal Health and Comparative Medicine, University of Glasgow, Glasgow, UK; 6 Wellcome Centre for Molecular Parasitology, University of Glasgow, Glasgow, UK; 7 Big Data Institute, University of Oxford, Oxford, UK

**Keywords:** Schistosomes, Kato-Katz, CCA, Mathematical models, Diagnostics, Trace readings

## Abstract

**Background:**

Schistosomiasis is a major socio-economic and public health problem in many sub-Saharan African countries. After large mass drug administration (MDA) campaigns, prevalence of infection rapidly returns to pre-treatment levels. The traditional egg-based diagnostic for schistosome infections, Kato-Katz, is being substituted in many settings by circulating antigen recognition-based diagnostics, usually the point-of-care circulating cathodic antigen test (CCA). The relationship between these diagnostics is poorly understood, particularly after treatment in both drug-efficacy studies and routine monitoring.

**Results:**

We created a model of schistosome infections to better understand and quantify the relationship between these two egg- and adult worm antigen-based diagnostics. We focused particularly on the interpretation of “trace” results after CCA testing. Our analyses suggest that CCA is generally a better predictor of prevalence, particularly after treatment, and that trace CCA results are typically associated with truly infected individuals.

**Conclusions:**

Even though prevalence rises to pre-treatment levels only six months after MDAs, our model suggests that the average intensity of infection is much lower, and is probably in part due to a small burden of surviving juveniles from when the treatment occurred. This work helps to better understand CCA diagnostics and the interpretation of post-treatment prevalence estimations.

**Electronic supplementary material:**

The online version of this article (10.1186/s13071-017-2580-z) contains supplementary material, which is available to authorized users.

## Background

Schistosomiasis is a major public health and socio-economic problem in many sub-Saharan African countries, with estimates of 240 million people infected [1]. The current strategy to control morbidity is based on mass drug administrations (MDA) with the anthelmintic drug praziquantel. However, this does not prevent reinfection, which can be very rapid, with prevalence often reported to quickly return to pre-treatment levels [2–5].

The main diagnostic tool currently used to evaluate *Schistosoma mansoni* prevalence and intensity of infection is the Kato-Katz thick smear technique (KK) [6], which measures the number of parasite eggs in one (or more) 41.7 mg smears taken from a stool sample. KK has a very high specificity (∼100%), and gives a quantitative measure of the level of infection, the egg count, which is positively correlated with disease burden. However, it has some disadvantages, including large day-to-day variation in egg outputs and readings [7–9] and low sensitivity at low levels of infection. This low sensitivity is due to fewer eggs being present at low intensities of infection and therefore less likely to be within the small sample of faeces [5, 10–13]. Eggs are still excreted after recent treatment, and are viable; with heavy excretions that can occur over a week post-treatment [5].

The “point-of-care” circulating cathodic antigen test (CCA) that measures schistosomal antigen in the urine is an alternative technique to evaluate presence of infection. These are regurgitated gut antigens from feeding worms, which might include juveniles. There are no soluble egg antigen (SEA) tests available to specifically measure egg presence. CCA therefore measures antigens in active worm infections that disappear from the body within 24 to 48 h of clearing the infection [14]. These assays are commercially available, and are now being used for countrywide mapping of the prevalence of schistosomiasis [15–17]. CCA has a higher sensitivity than KK, particularly at low intensity of infection, but there are some indications that specificity might not be 100% [5, 13, 18, 19]. One of the challenges of estimating specificity is the lack of an accurate “gold standard” against which CCA can be evaluated [19–21]. CCA possesses other limitations, such as how to interpret “trace” results, which is believed to represent a large proportion of true positives although again remains under debate and may vary across differing endemicity level areas, with urine concentration and between test readers [21, 22].

Despite several community- and national level-based studies, the relationship between KK and CCA diagnosis is still not well understood. Latent Markov models [23], latent class analyses [13, 24] and generalized linear mixed models [15] have been used to help elucidate this and address the issues associated with “trace” readings. However, these studies have focused on data pre-treatment and these relationships may become more complex and particularly important after recent treatment.

In this paper, we combined the information from KK and CCA pre-treatment, one month post- and six months post-treatment in a high endemic *S. mansoni* area, to create a mathematical model of the relationship between the two diagnostics at an individual level. We fitted this model using a Bayesian framework and estimated true underlying schistosome prevalence. We then used the model to simulate populations and sample KK and CCA diagnostics, with the aim of comparing the error in the estimation of prevalence using either CCA or KK (with different numbers of stool samples analysed).

## Methods

### Data

The dataset used in this manuscript was sourced from a longitudinal study in three primary schools in Mayuge District, Uganda, a highly endemic area on the shores of Lake Victoria [5]. The students, between the ages of 6 and 12, were sampled at baseline (before treatment), one month after treatment and six months after treatment. At each sampling time, KK was performed in duplicate over three days as well as one CCA from a single urine sample. The number of individuals tested was 364, although there are missing data in some entries, with some individuals not having all six KK results, these can be inferred by the model (see below). More information on the sample collection and processing details is outlined by Lamberton et al. [5].

### Base model

We created a mathematical model of *S*. *mansoni* infection in humans, where we assumed a gamma distribution of true intensity of infection across the infected population combined with a negative binomial distribution of error in these estimations at each time-point, in a similar fashion to the zero-inflated model developed in Atlija, Prada et al. [25], see Additional file 1 for a detailed model description. This framework allows discrimination between infected and uninfected individuals, and we can estimate infection status based on the information we have from each individual’s diagnostics results. Individuals with undetectable levels of infection can potentially be assigned to either the infected or uninfected group. We can therefore define individuals’ infectious status as

$$ {Status}_{i,t}=\left\{\begin{array}{ll}0& \mathrm{uninfected}\  \mathrm{or}\  \mathrm{undetectable}\  \mathrm{infection}\\ {}1& \mathrm{infected}\end{array}\right. $$

where *Status* = 0 means that the individual is uninfected or with undetectable levels of infection, while *Status* = 1 means that the individual is infected (at either detectable or undetectable levels), for each individual *i* at time *t*. The true prevalence at each time-point *t*, is the proportion of individuals with *Status* = 1, henceforth referred to as *P*_*t*_.

Each infected individual has an intensity of infection, *λ*_*i*,*t*_, drawn from a gamma distribution of intensity across the population, with shape, *s*_*t*_, and rate, *r*_*t*_, parameters being estimated in the model at each time-point *t*,

$$ {\lambda}_{i,t}= Gamma\left({s}_t,{r}_t\right) $$

Kato-Katz, like any other method for obtaining faecal egg counts is susceptible to measurement error, particularly at low levels of infection. Unlike previous work where a Poisson distribution was used [25], we instead employed a negative binomial distribution, as the variance in the counts is higher than the mean [26, 27], with an over-dispersion parameter *ω*. Moreover, uninfected individuals by definition do not have schistosomes, therefore cannot have KK counts different from zero; based on the individuals’ status estimated above, the KK diagnostic for individual *i*, at time *t*, for the repeated count *c* would be:

$$ {KK}_{i,t,c}=\left\{\begin{array}{cc}0& {\mathrm{If}\  \mathrm{Status}}_{\mathrm{i},\mathrm{t}}=0\\ {} NegBinomial\left({\lambda}_{i,t},\omega \right)& {\mathrm{If}\  \mathrm{Status}}_{\mathrm{i},\mathrm{t}}=1\end{array}\right. $$

Similarly, the individual’s status will determine possible CCA test outcomes and since the CCA test used in the field is a semi-quantitative diagnostic, with possible values of negative (−), *trace*, positive (+), double positive (+ +) and triple positive (+ + +), we converted them into 0, 1, 2, 3 and 4, respectively. For infected individuals, we calculated${P}_{i,t}^{CCA}$, which is a value between zero and one, that depends on the estimated intensity of infection *λ*_*i*, *t*_, via a logistic function with model parameters *k*_1_ and *k*_2_ being estimated

$$ {P}_{i,t}^{CCA}=\raisebox{1ex}{$1$}\!\left/ \!\raisebox{-1ex}{$1+{e}^{-{k}_1\left({\lambda}_{i,t}-{k}_2\right)}$}\right. $$

We then used the${P}_{i,t}^{CCA}$value to simulate the CCA diagnostic outcome with a binomial distribution of *n* = 4, such that highly infected individuals are more likely to be diagnosed positive [or (+ +)/(+ + +)]. On the other hand, uninfected individuals, if the specificity of the diagnostic was 100%, should have negative CCA. Because the specificity is lower, we allowed for some uninfected individuals to have *trace* results in the diagnostic, with a probability*P*_*Tr*_; the CCA diagnostic would therefore be:

$$ {KK}_{i,t,c}=\left\{\begin{array}{cc} Bernouilli\left({P}_{Tr}\right)& {\mathrm{If}\  \mathrm{Status}}_{\mathrm{i},\mathrm{t}}=0\\ {} Binomial\left(n=4,p={P}_{i,t}^{CCA}\right)& {\mathrm{If}\  \mathrm{Status}}_{\mathrm{i},\mathrm{t}}=1\end{array}\right. $$

This particular framework assumes that KK specificity is 100%, and thus uninfected individuals must have zero eggs, as well as limiting the possible CCA results for uninfected individuals to either (−) or *trace*. The true prevalence estimate (i.e. proportion of infected individuals, *P*_*t*_) can then be compared to the KK and CCA prevalence from the raw data.

The model was fitted to all three time-points concurrently. We forced the mean intensity of infection (mean of the gamma distribution) for the time-point one month after treatment (*t* = 2) to be lower than at baseline (before treatment, *t* = 1) and at six months post-treatment (*t* = 3) such that

$$ \frac{s_2}{r_2}<\frac{s_1}{r_1},\frac{s_3}{r_3} $$

Another model assumption that we made was that the relation between infection level and the CCA test result is independent of the prevalence, this assumes that test interactions only occur within an individual at any given time, and thus we fitted one *k*_1_ and *k*_2_ parameter for all three time-points. We also assumed a single over-dispersion parameter *ω* across all time-points.

The model was run in a Bayesian framework using a *Gibbs* sampling package in R [28] (“*jags*” [29] and “*runjags*” [30]), with two independent chains, a ‘burn-in’ period of 1000 iterations and 10,000 samples. Uninformative priors where used. Convergence was assessed by visual examination of the trace plots and the Gelman-Rubin statistic. The run includes all 364 individuals, with up to six KK samples and one CCA value each for every time-point, with the missing KK data inferred by the model. We assessed the goodness-of-fit of the model by comparing draws from the posterior distribution to the data, see Additional file 1.

### Sampling from the model fit

After the model fitting, as described in the previous section, we obtained a posterior distribution for each of the fitted parameters (shapes and rates of the gamma distribution at each time point, *ω*, *k*_1_ and *k*_2_ and *P*_*Tr*_). We explored the relationship between the estimated true prevalence and the average infection level, as prevalence on average decreases at lower infection levels. This relationship is not linear, and we sampled without extrapolating beyond the data, correlated values of average infection level and prevalence, see Additional file 2. These values are used to generate simulated populations, and perform KK (two samples) and CCA tests on those populations. We compared how KK and CCA prevalence changes as true prevalence varies. We also explored the error in the estimation of prevalence using one to six KK and CCA, assuming trace results as either positive (CCA+) or negative (CCA-).

## Results

**Fig. 1:**
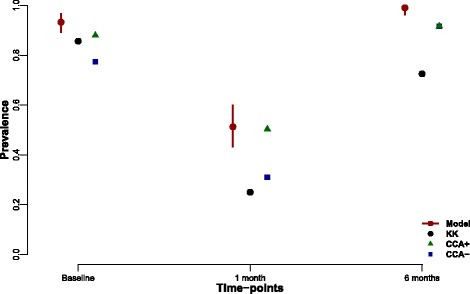
Prevalence comparison between raw data and model prediction. Point estimates are the predicted values based on the raw data, black circles represent estimated prevalences using Kato-Katz, while the green triangles and blue squares are the estimates using a circulating cathodic antigen diagnostic, assuming trace results as positive (CCA+) and negative (CCA-), respectively. Red point with credible interval is the prevalence estimated by the model. Individual diagnostics prevalence estimates are generally lower than model predicted values combining both diagnostics

We compared prevalence estimations from the raw data (either KK or CCA) with our model estimation of true prevalence (Fig. 1). In general, both KK and CCA underestimate the prevalence of schistosomiasis in our population compared to the model. It is important to note that trace results in the CCA test can be either considered infected (Fig. 1; CCA+, green triangle) or uninfected (Fig. 1; CCA-, blue square). Using trace results in the CCA as positive improves the estimation of prevalence over trace results as negative for *S. mansoni* in this high endemicity community, and is a better estimator than KK at all time points. We estimated the proportion of truly uninfected individuals that have trace CCA results (rather than negative) to be around 15%, albeit our credible interval ranges from 4 to 27%.

**Fig. 2:**
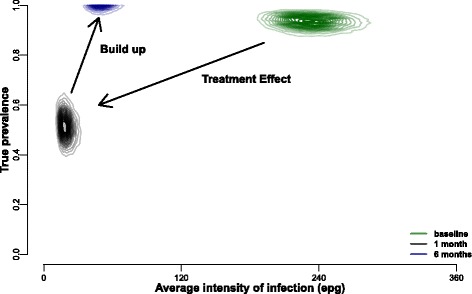
Posterior distribution of average intensity of infection vs true prevalence. Each colour represent a different time point. At baseline (green) both prevalence and intensity of infection are high; one month post-treatment (black) both prevalence and infection are low; six months post-treatment (blue) the prevalence is similar to baseline, but the average population intensity of infection is still relatively low. This suggests that prevalence recovers faster in the population than intensity of infection, which builds up more slowly. The intensity of infection is shown in eggs per gram (epg) for clarity, however, the raw data are used in the model

The posterior draws from the model fitting were used to compare average estimated underlying intensity and prevalence of infection in this community (Fig. 2). At baseline before treatment, the estimated true prevalence was high, as expected, and so was the average intensity of infection in the population (green area). One month after treatment, both the prevalence and average intensity of infection have drastically decreased (black area). However, six months after treatment (blue area), prevalence levels were similar to pre-treatment values, but the average intensity of infection was lower than at baseline, and moderately higher than one month after treatment.

**Fig. 3:**
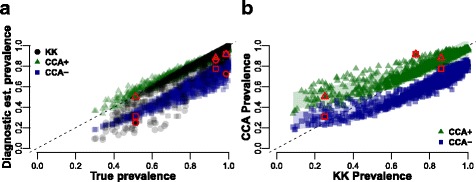
Comparison between diagnostic estimates and true prevalence for simulated data. Colours represent the different diagnostics. Double KK estimates are coloured black (circles), CCA with trace results as positives (CCA+) are represented by green triangles, while CCA with trace as negative (CCA-) are blue squares. Symbols coloured red are the real data from the diagnostics for the average estimated true prevalence (Baseline and one and six months post-treatment). **a** Comparison of the different diagnostics and true prevalence illustrating that KK and CCA- always underestimate true prevalence, while CCA+ overestimates prevalence at low/medium prevalences. **b** Comparison of CCA diagnostics (CCA+ and CCA-) and KK diagnostics (two samples) showing a non-linear relationship as prevalence changes

We then interpolated across a range of infection levels to assess how the estimation of CCA and KK prevalence varies as true prevalence changes (Fig. 3a), and the relationship between both diagnostics (Fig. 3b). In Fig. 3a, the dotted diagonal indicates a diagnostic yielding the true prevalence, with points under the diagonal underestimating true prevalence, while the opposite is true for the points above the diagonal. As true prevalence increases, the difference in the prevalence estimation error between CCA and double KK diagnostics decreases. Only at high prevalence levels, from approximately over 70–80%, we see KK (Fig. 3; black) giving in general higher prevalence estimates (and therefore closer to the true prevalence) than CCA assuming trace readings as negative (Fig. 3; CCA-, blue). Whereas, if we consider a CCA trace reading as a positive result, CCA diagnostics would generally underestimate true prevalence above 85%, but will otherwise tend to overestimate prevalence at lower levels of true prevalence (Fig. 3; CCA+, green). In Fig. 3b we can see the non-linear relationship between both diagnostics. CCA+ diagnostics will rapidly overestimate prevalence compared to KK as prevalence decreases (i.e. above the diagonal). On the other hand, CCA- estimates only give higher estimated prevalences than KK at low levels of prevalence, with both diagnostics underestimating true prevalence (Fig. 3). We did not simulate values below ∼35% true infection prevalence, as this was the lower value of estimated true prevalence in our data, corresponding to 1 month post-treatment (Fig. 3). It is important to note that prevalence estimated with either CCA- or KK always underestimated true prevalence in our simulations.

**Fig. 4:**
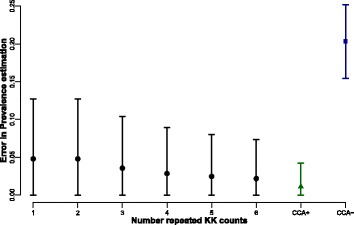
Error in prevalence estimation based on simulated data. Mean and standard deviation across all simulated points. Error decreases as the number of repeated Kato-Katz measures increases but CCA assuming trace as positive (CCA+) has an overall smaller error in prevalence estimation. This suggests that multiple KK will improve precision, but not enough compared to CCA+, particularly at low levels of prevalence

In the simulated population, we also mimicked different repeated KK test results, from one to six samples (informed by the data) and compared the error in the estimation of true prevalence (Fig. 4). The error in true prevalence estimation slightly decreased as more samples were examined by KK. However, CCA assuming trace results as positive (Fig. 4; green) had the smallest error in prevalence estimation, smaller than even six repeated KK. Of particular note is that using CCA but assuming trace readings were negative (Fig. 4; blue), had a significantly larger error than even a single KK across our simulated populations.

## Discussion

We have performed an analysis to compare prevalence estimates using one to six repeated KK and a single CCA test, to understand how they differ from estimated true prevalence values. This is particularly important as current public health policy decisions are based on prevalence by KK [31], and the estimates obtained using CCA are often higher and will have a different degree of bias. Moreover, since CCA is now being used in some countries to map schistosomiasis, it is crucial to understand how to interpret these new results so that control strategies can be updated accordingly and additional drug treatment requests and production be set in motion [22].

Kato-Katz smears have historically been vital for diagnosis and policy decisions and remain extremely useful at high levels of prevalence. This is because a high prevalence is associated with an average high intensity of infection. However, many highly endemic countries have been implementing mass control programmes for over a decade now and infection intensities in several regions, but not all, are now lower. Low sensitivity of the diagnostic test at low infection levels is now becoming an important issue. As shown in Fig. 1, the KK estimate of prevalence is significantly lower six months post-treatment than at baseline (∼10% lower estimate), even though the estimated true prevalence is the same. This becomes apparent when looking at the average intensity of infection estimates which show much lower intensities six months post-treatment (Fig. 2). CCA, on the other hand, is generally a better estimator for true prevalence, particularly when assuming that trace results are positive, although this will lead to overestimation of prevalence at low true prevalences (Fig. 3a; green). This could be in part because even if a proportion of the trace results belong to uninfected individuals (we estimate it to be 15% on average), at a population level it helps minimize the underestimation of true prevalence inherent in the diagnostic (Fig. 4).

The relationship between prevalence estimates from CCA and KK as true prevalence changes is not linear (Fig. 3b) and in general, CCA is a better estimator of prevalence, particularly at low prevalences. The relationship we have estimated could be used to compare historical KK prevalence estimates to more contemporary, often higher CCA estimates. This is particularly helpful for programmes which have switched from KK to CCA and need to make informed public health decisions. However, it is still unclear exactly how the intensity of infection and prevalence increases after treatment over a short timescale, and more time-points are needed between one and six months post-treatment. In our approach we did not extrapolate beyond our data and therefore assumed the correlation between prevalence and intensity to be maintained.

It is also important to note that observing high prevalences (or even pre-treatment prevalence levels) a few months after treatment does not necessarily mean that intensities of infection are the same as before treatment (Fig. 2). With KK, we would potentially estimate a lower prevalence after treatment, because the average egg count in the population will be lower. However, using CCA as a diagnostic tool instead means that we would recover a better estimation of prevalence (i.e. a high prevalence), but we would miss the fact that the average intensity of infection is in fact reduced. A biological justification for this could be that praziquantel treatment has a higher efficacy in adult schistosomes and a reduced effect on juveniles [32, 33]. Therefore a few months after treatment, we would expect to see the juveniles becoming adults, but the overall infection load may still be low, due to a lower schistosome adult populations and only six months of new parasite exposure. This varying relationship between diagnostic test and accuracy in detecting prevalence versus intensity is important because public health guidelines for control are focused on prevalence data, whilst morbidity is positively correlated with infection intensity. Therefore, there is a potential disconnect between the prevalence indicators which WHO use for treatment guidelines and the intensity indicators which the WHO have as goals for morbidity control, with each of them also being affected by the choice of diagnostic test.

Future genetic studies may help elucidate the origin of the parasite eggs and/or antigens post-treatment, to decipher whether antigens are from new infections or surviving juveniles, which were not affected by the treatment, or whether they are from adult worms surviving the standard treatment and therefore resistant to the currently used drug praziquantel. In addition, the true meaning of a “trace” reading, and the absence of a “gold standard”, may become more important after recent praziquantel treatment than our current model can explain. Correlations held at low intensities pre-treatment in low endemicity area, may not hold for similar low intensities (measured by KKs) post-treatment. These two situations, which may have similar mean eggs per gram values measured by KKs alone could reflect vastly different biological scenarios. Recent praziquantel treatment may interact with parasite juvenile and adult worm numbers, and egg production and clearance. All of these factors, plus the rate of continued parasite exposure and reinfection, strongly linked to endemicity and force of infection, may affect egg and/or antigen excretion at follow up. This in turn affects KK and/or CCA readings, blurring any pre-treatment correlations between KK and CCA outputs even further. This may in part explain why point estimates in Fig. 1 are more tightly clustered between diagnostic techniques than post-treatment.

## Conclusions

We have shown the relationship between the two most commonly used diagnostic tools for *S. mansoni* infection, an egg-based and an antigen-based diagnostic. Having multiple diagnostics for a particular disease is becoming more common, but connecting the alternative diagnostics can prove challenging. In neglected tropical diseases, new studies are being done in this scope, such as the work by Irvine et al. [34] in lymphatic filariasis diagnostics. Understanding the interactions between the different diagnostic tools is important, particularly for diseases such as schistosomiasis that have ambitious targets for control and elimination by 2020. This study improves our understanding of these interactions and how to interpret prevalence and intensity measures of *S. mansoni* infection pre- and post-treatment.

## Additional files

Additional file 1: Detailed model description. Document explaining and justifying all model assumptions. Posterior distributions from the analysis are also shown, as well as the fit to the data. (PDF 2476 kb)

Additional file 2: Sampling interpolation methodology. Methodology to sample correlated infection levels and average intensity of infection. (PDF 82 kb)

Additional file 3: Dataset. (CSV 21 kb)

## Abbreviations

CCA: Circulating cathodic antigen
CCA +: Trace as positive CCA
CCA-: Trace as negative CCA
KK: Kato-Katz
MDA: Mass drug administration
