# Supplementary material for: Understanding the relationship between egg- and antigen-based diagnostics of Schistosoma mansoni infection pre- and post-treatment in Uganda
Source: Parasit Vectors. 2018 Jan 8;11:21. doi: 10.1186/s13071-017-2580-z (PMC5759883; doi:10.1186/s13071-017-2580-z)
Supplement: Supplementary file 1 — Detailed model description. Document explaining and justifying all model assumptions. Posterior distributions from the analysis are also shown, as well as the fit to the data. (PDF 2476 kb) [file 13071_2017_2580_MOESM1_ESM.pdf]

Understanding the relationship between egg- and antigen-based diagnostics of *Schistosoma mansoni* infection pre- and post-treatment in Uganda

Additional file 1 - Detailed model description

J. M. Prada, P. Touloupou, M. Adriko, E. M. Tukahebwa, P. H. L. Lamberton and T. D. Hollingsworth

December 4, 2017

The model is based on a mechanistic understanding of the relationship between an individual's true infection status (which is unobserved), their intensity of parasite infection (also unobserved) and their detectability by the different diagnostics. We first describe the model for their true infection status and then the model for Kato-Katz (KK) and Circulating Cathodic Antigen (CCA). This supplementary material extends the model description from the main manuscript, with clarifications for the choice of distributions and other model parameters, as well as more details on the model posteriors and fit to the data.

1 Individual's status

We need a model with the flexibility to differentiate between individuals that are clear of infection (which could be due to lack of exposure or recent treatment) and individuals who are still infected (who might or not have received treatment). The model developed here follows the same principles of the zero-inflated model from Atlija, Prada *et al.* [1]. We first introduce the notation for an individuals true infection status and give an overview of the fitting approach. We define the individual's status, with the following formulation,

$$Status_{i,t} = \begin{cases} 0 & \text{uninfected or undetectable infection} \\ 1 & \text{infected} \end{cases} \tag{1}$$

for individual i at time t. Note here each time-point is treated independently, and we don't track individuals' status from one time-point to the next.

Heuristically, in order to infer an individual's infection status, we perform a series of related simulations (Gibbs sampler) in which each individual is randomly allocated the status; to be infected (status=1) or uninfected (status=0). If a simulation gives a "good" fit to the data then we keep that simulation. A good fit is defined by how well the model simulated diagnostic tests (see further description of the model, below) compare to the observed diagnostic test results for each individual. Then we randomly shuffle people's status again. Over many iterations of this process, a particular individual will be more frequently infected or uninfected. This allows us to have an estimate of the probability that an individual is infected or not. At a population level this yields a distribution of probabilities between 0 and 1 at each time-point, Figure S1.1.

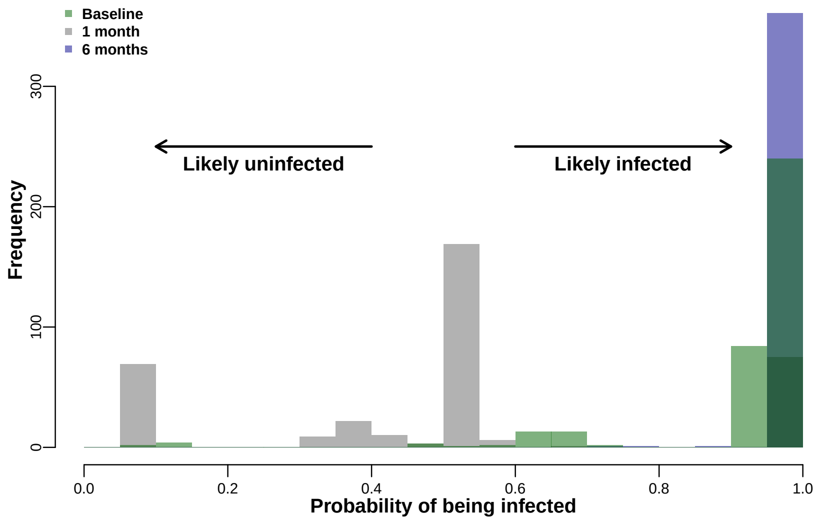

Figure S1.1: Histogram of probabilities of being infected at the three different time-points. At baseline (green) and 6 months post-treatment (blue), most individuals are very likely to be infected. 1 month post-treatment (grey), some individuals are very likely to be infected, some a very likely to be uninfected and for some it is hard to say.

2 Intensity of Infection

The intensity of infection across a population (particularly for parasitic diseases such as schistosomiasis) is generally modelled using a negative binomial distribution. This distribution is equivalent to a Gamma-Poisson process; that is, a gamma distribution of "true" intensity of infection (henceforth $\lambda_{i,t}$ for individual i at time t) and a Poisson distributed counting error. We hence define $\lambda_{i,t}$,

$$\lambda_{i,t} = Gamma(s_t, r_t) \tag{2}$$

with s_t and r_t the shape and rate parameters respectively, which are estimated by the model.

3 Modelling Kato-Katz

The Kato-Katz counts are generated differently depending on the individuals' status. If $Status = 0$, the Kato-Katz have to be zero; this assumption implicitly assumes that the specificity is 100% (i.e. if the individual is uninfected, it cannot have positive egg counts). If $Status = 1$, the Kato-Katz are generated based on the intensities of infection ($\lambda_{i,t}$, above) with some counting error. In previous work ([1]), a Poisson error was enough, however, these KK data are from double measures three consecutive days, therefore the over-dispersion will be higher than Poisson. In line with previous work by de Vlas *et al.* [2], a negative binomial was used for the counting error instead of a Poisson. This means that at a population level, the KK counts are generated through a Gamma-Negative Binomial process. In summary,

$$KK_{i,t,c} = \begin{cases} 0 & \text{If } Status_{i,t} = 0 \\ NegBinomial(\lambda_{i,t}, \omega) & \text{If } Status_{i,t} = 1 \end{cases} \tag{3}$$

for individual i, at time t, for repeat c, with ω being the over-dispersion parameter of the negative binomial which is estimated by the model.

4 Modelling CCA

The CCA test used in the field is a semi-quantitative diagnostic, with possible values of negative ($-$), *trace*, positive ($+$), double positive ($++$) and triple positive ($+++$). Intuitively we can assume that the higher the intensity of infection, the more likely we are to test positive. This increase could be slow and linear or could be very sharp at a certain threshold (similar to a step function). To allow flexibility, we modelled this relationship with a logistic function between zero and one, such that as the intensity of infection ($\lambda_{i,t}$) increases, the probability of testing CCA positive also increases. We constrained this logistic function to be between zero and one, so that the outcomes can be used as proportions, changing these limits would only re-scale the function, and will not alter results.

$$P_{i,t}^{CCA} = \frac{1}{1 + e^{-k_1(\lambda_{i,t} - k_2)}} \quad (4)$$

where k_1 and k_2 are estimated by the model. Note that for a given value of k_1 and k_2, each individual i will have a different P^{CCA} based on their intensity of infection λ, at time t.

If the diagnostic was a simpler test (positive/negative), then this probability could simply be used in a Bernoulli trial. Note that the analysis carried in here could be done this way by collapsing positive (+), double positive (++) and triple positive (+ + +) into the same category and allocating *trace* results to either positive or negative. To maintain the resolution of the data, we decide to use a binomial distribution with four draws, which will output a value between zero and four. An additional advantage is that the densities peak at the extremes (i.e. low $P_{i,t}^{CCA}$ will very likely test zero - negative, Figure S1.2). The disadvantage is that this distribution is relatively "flat" for probabilities around 50%, which means that individuals with those $P_{i,t}^{CCA}$ could test a wide range of values in the CCA diagnostic, Figure S1.2.

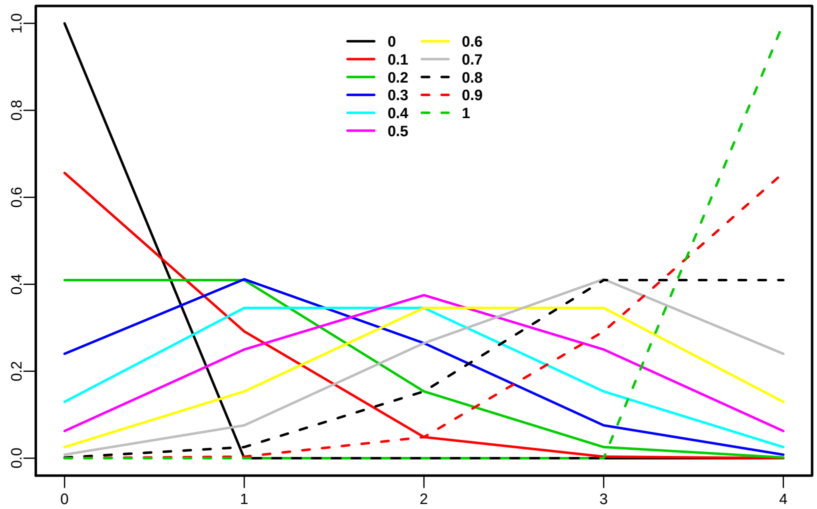

Figure S1.2: Binomial distribution with size 4 for different probability values

Individuals with $Status = 0$ are in our model not infected, but to allow some flexibility with the diagnostic, we used a Bernouilli distribution for the antigen-based test. This allows individuals with CCA diagnostic either negative or *trace* could to potentially be identified as not infected. Note that strictly speaking it does not represent the specificity, as false positives could potentially be (+), (++) or (+ + +) as well, but it does account for part (we believe most) of it. We summarize the equations for different individuals' status as,

$$CCA_{i,t} = \begin{cases} Bernoulli(P_{Tr}) & \text{If } Status_{i,t} = 0 \\ Binomial(n = 4, p = P_{i,t}^{CCA}) & \text{If } Status_{i,t} = 1 \end{cases} \quad (5)$$

where P_{Tr} represents the proportion of uninfected individuals ($Status = 0$) that have a *trace* result in the CCA test, this value is estimated by the model.

5 Additional assumptions

To increase the power of the data, we are fitting all three time-points concurrently and assume that some model parameters are time independent. These are the parameters without a t subscript; the logistic function parameters for the CCA diagnostic, k_1 and k_2, which implies that the infection level and the CCA test result is independent of prevalence; the proportion of uninfected individuals with *trace* results in the CCA, P_{Tr}, which suggests that this error is prevalence independent; and the over-dispersion parameter in the Kato-Katz method, ω, which indirectly assumes dependence on intensity of infection.

Moreover, since individuals are treated after the first time point (baseline), we force in the model the mean intensity of infection one month post treatment to be the lowest of the three time-points. The mean intensity of infection in this model is represented by the mean of the Gamma distribution, which is calculated by dividing the shape by the rate, therefore,

$$\frac{s_2}{r_2} < \frac{s_1}{r_1}, \frac{s_3}{r_3} \tag{6}$$

where $t = 1$ is baseline, $t = 2$ is one month post-treatment and $t = 3$ is six months post-treatment.

6 Posterior distributions and model fit

The model was set up in a Bayesian framework in R [3] using JAGS (Just Another Gibbs Sampler, [4]) with the "runjags" package [5]. We ran two independent chains, which were well mixed for all model parameters, we therefore used the combined chain as the posterior, see figures below.

The first parameter of interest are the zero-inflation parameters, which are essentially the prevalences at the different time-points. The proportion of individuals belonging to the zero distribution ($status = 0$) is 1-Prevalence, Figure S1.3.

Another parameters of interest are the shapes and rates that define the Gamma distributions at the three time-points. We show in Figure S1.4 the Gamma distributions with the median shapes and rates parameters at each time-point (solid lines). Furthermore, we generated 1000 Gamma distributions by drawing shapes and rates randomly from the posterior distribution, Figure S1.4 - shaded areas.

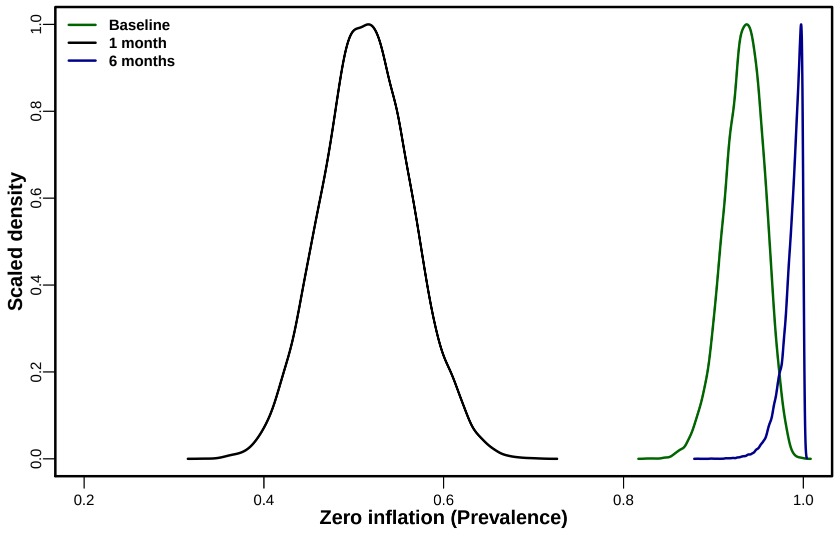

Figure S1.3: Prevalence estimates at the three time-points, baseline (green), one month (black) and six months (blue) post-treatment. y axis is the scaled density calculated as density/max(density).

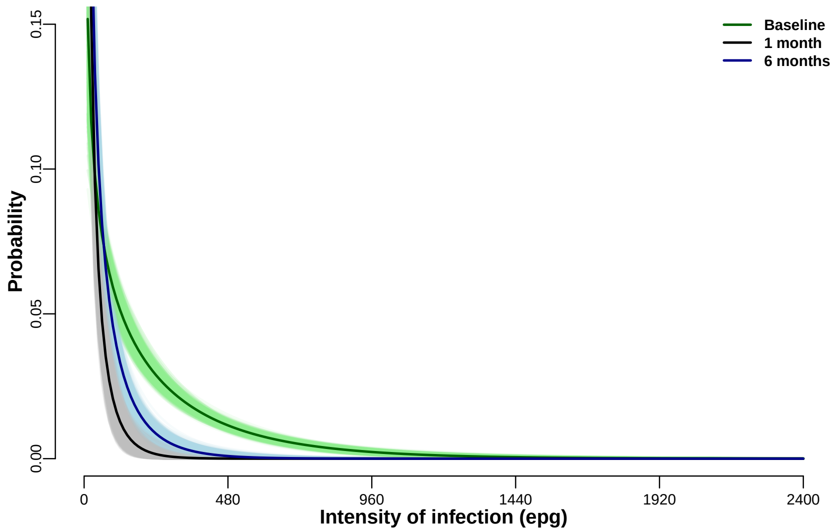

Figure S1.4: Gamma distributions at the three time-points. Solid lines represent the distribution with median shape and rate values. Shaded areas area generated Gamma distributions using parameters drawn from the posteriors. Intensity of infection (x axis) has been scaled to eggs per gram by multiplying the raw counts by 24. This was done for illustrative purposes only, raw counts were used throughout the analysis.

The next parameters analyzed were k_1 and k_2, which define the shape of the logistic function that we used to relate the intensity of infection to a probability, $P_{i,t}^{CCA}$, which we need to generate the CCA diagnostic results. Similar to the previous plot, in Figure S1.5 the solid line describes the function with the median values for k_1 and k_2 while the shaded area are the 1000 logistic curves generated by drawing values for k_1 and k_2 from their posterior distributions.

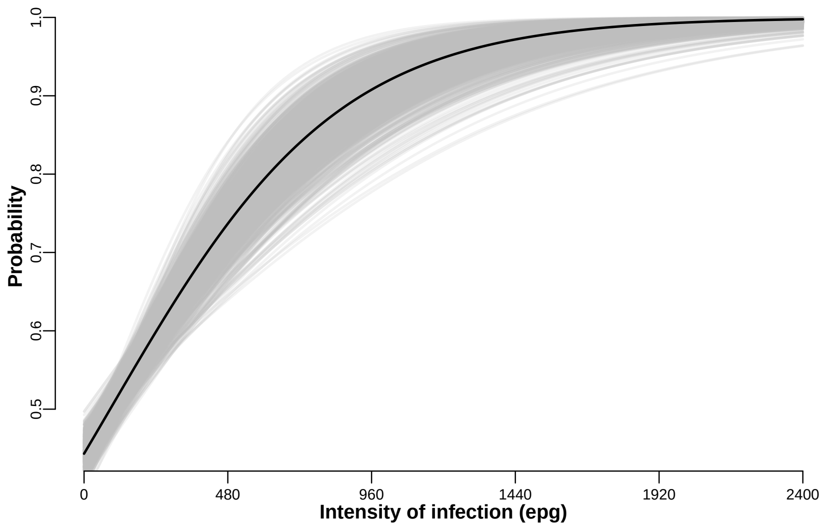

Figure S1.5: Logistic function to link the intensity of infection with a probability, $P_{i,t}^{CCA}$, which is later used to simulate the CCA diagnostic results at a population level. Intensity of infection (x axis) has been scaled to eggs per gram by multiplying the raw counts by 24. This was done for illustrative purposes only, raw counts were used throughout the analysis.

In our model we assumed that uninfected individuals ($status = 0$) could have either negative CCA or *trace*. This was done to allow for potential cross-reactivity. In Figure S1.6 we show the density plot of the posterior distribution of this proportion of individuals testing *trace* while being uninfected, P_{Tr}. This posterior is quite broad, with values ranging from zero to 0.4, with the peak around $0.15 - 0.2$.

The last parameter of interest in the model is the over-dispersion of egg counts. As described by de Vlas *et al.* [2], a negative binomial is the most appropriate distribution to model the error in the Kato-Katz methodology, particularly when there is day-to-day variation to take into account, as in our case. The negative binomial is parametrized by a p and r parameter, and we calculate them from the distribution mean (given by $\lambda_{i,t}$) and the over dispersion ω, Figure S1.7.

$$\begin{aligned} p &= \frac{\omega}{\lambda_{i,t}+\omega} \\ r &= \omega \end{aligned} \tag{7}$$

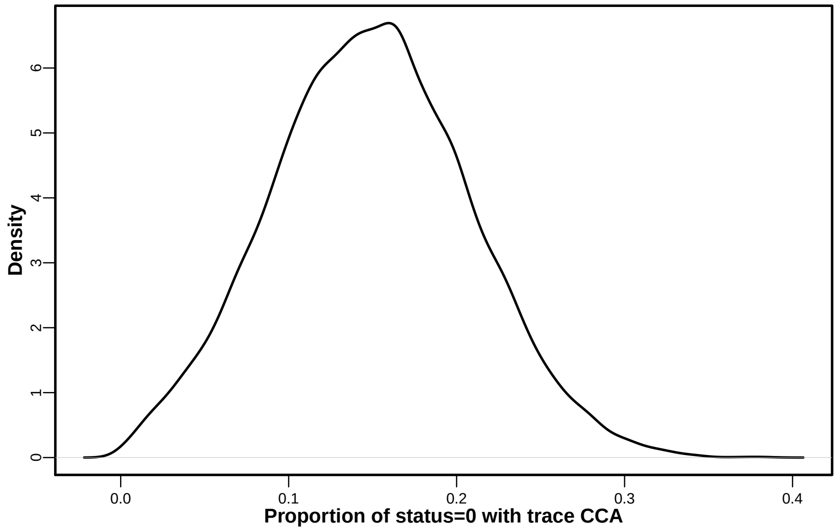

Figure S1.6: Proportion of uninfected individuals ($status = 0$) that test $trace$ instead of ($-$) for CCA, P_{Tr}. Note that this proportion does not go below zero, and it is an artifact from the smoothing.

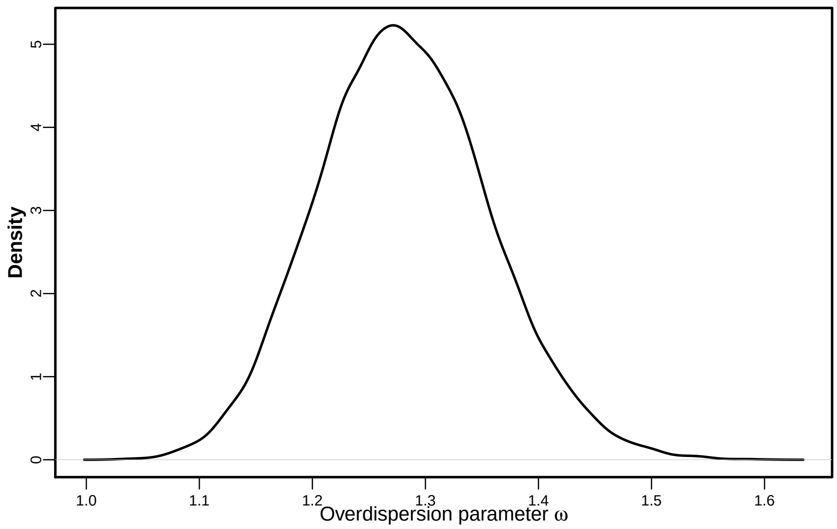

Figure S1.7: Over dispersion parameter ω.

Drawing from the posterior distribution for all these parameters, we generated KK and CCA diagnostics for a simulated population to verify that we could reproduce the data. We were particularly interested in reproducing the relationship between CCA and KK. We generated 200 populations and calculated the credible interval for the range of Kato-Katz values for each CCA category, $(-)$, *trace*, $(+)$, $(++)$ and $(+++)$. Our simulated populations generally span a similar range than the data, Figure S1.8. Because we are pooling the 200 populations, we expect the confidence intervals to be "shorter" than the data, due to the averaging effect. However, we can generate individuals with higher KK than the data for all five CCA categories. The other feature to note, is the apparent "dip" in KK for individuals with *trace* CCA, however, this is due in the data to one individual with negative CCA and a relatively high KK for that group, Figure S1.8.

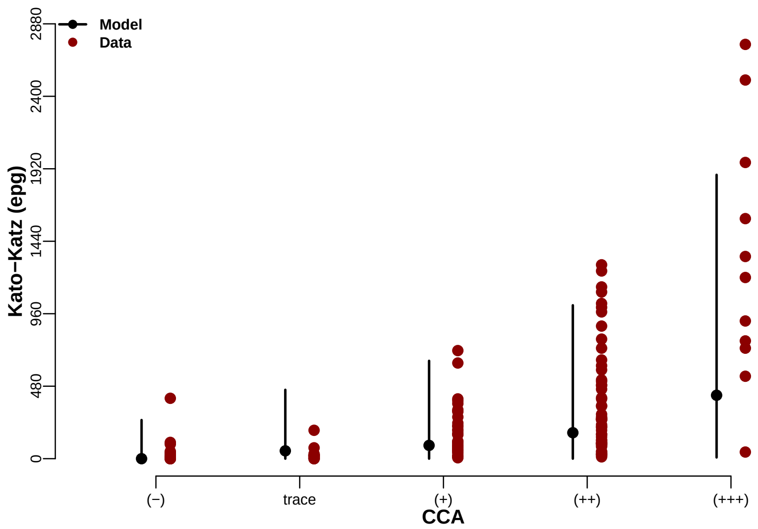

Figure S1.8: Comparison of Kato-Katz ranges for each CCA category (result in the diagnostic). KK counts (y axis) have been scaled to eggs per gram by multiplying the raw counts by 24. This was done for illustrative purposes only, raw counts were used throughout the analysis. Red dots are the data, while the black dot represents the model prediction (median) and line for the confidence intervals (95%).

References

[1] M. Atlija, J. M. Prada, B. Gutiérrez-Gil, F. A. Rojo-Vázquez, M. J. Stear, J. J. Arranz, and M. Martínez-Valladares, “Implementation of an extended zinb model in the study of low levels of natural gastrointestinal nematode infections in adult sheep,” *BMC Veterinary Research*, vol. 12, no. 97, 2016.

[2] S. J. De Vlas, B. Gryseels, G. J. Van Oortmarssen, A. M. Polderman, and J. D. Habbema, “A model for variations in single and repeated egg counts in Schistosoma mansoni infections,” *Parasitology*, vol. 104 (Pt 3), pp. 451–460, Jun 1992.

[3] R Core Team, *R: A Language and Environment for Statistical Computing.* R Foundation for Statistical Computing, Vienna, Austria, 2016.

[4] M. Plummer, “JAGS: A program for analysis of Bayesian graphical models using Gibbs sampling,” in *Proceedings of the 3rd International Workshop on Distributed Statistical Computing*, 2003.

[5] M. J. Denwood, “runjags: An R package providing interface utilities, model templates, parallel computing methods and additional distributions for MCMC models in JAGS,” *Journal of Statistical Software*, vol. 71, no. 9, pp. 1–25, 2016.
