# Supplementary material for: Understanding the relationship between egg- and antigen-based diagnostics of Schistosoma mansoni infection pre- and post-treatment in Uganda
Source: Parasit Vectors. 2018 Jan 8;11:21. doi: 10.1186/s13071-017-2580-z (PMC5759883; doi:10.1186/s13071-017-2580-z)
Supplement: Supplementary file 2 — Sampling interpolation methodology. Methodology to sample correlated infection levels and average intensity of infection. (PDF 82 kb) [file 13071_2017_2580_MOESM2_ESM.pdf]

Understanding the relationship between egg- and antigen-based diagnostics of *Schistosoma mansoni* infection pre- and post-treatment in Uganda

Additional file 2 - Sampling interpolation methodology

Joaquín M. Prada *et al.*

December 4, 2017

The model we developed was fitted to three time points, at baseline, one and six months post-treatment. When we plot the posterior distribution obtained for those time points for the estimated true prevalence and average intensity of infection, we obtain three non-overlapping areas, Figure S2.1.

To sample a range of possible prevalence values without extrapolating beyond the data, we needed to maintain the relationship between prevalence and average intensity of infection. Because all three time-points yield non-overlapping posteriors, we sampled the prevalence and average intensity of infection from the convex hull (i.e. the area between the three defined posteriors in Figure S2.1). However, the intensity of infection in the population was modelled with a gamma distribution, so we need to sample not only a mean, but also a variation, while maintaining the correlation between the two, so that biologically "reasonable" gamma distributions are defined.

We therefore first sampled from a bivariate uniform distribution correlated values of mean and rate of the gamma distribution, using the correlation obtained from the posteriors (-0.78). We then sampled uniformly a value of prevalence that is contained within the convex hull (area) formed by the three time-points, an example is shown in Figure S2.2.

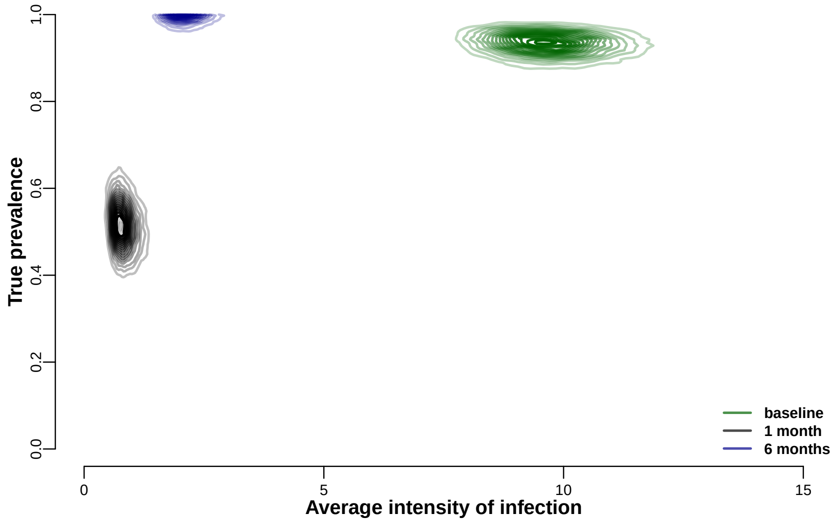

Figure S2.1: Posterior distribution of average intensity of infection vs estimated true prevalence.

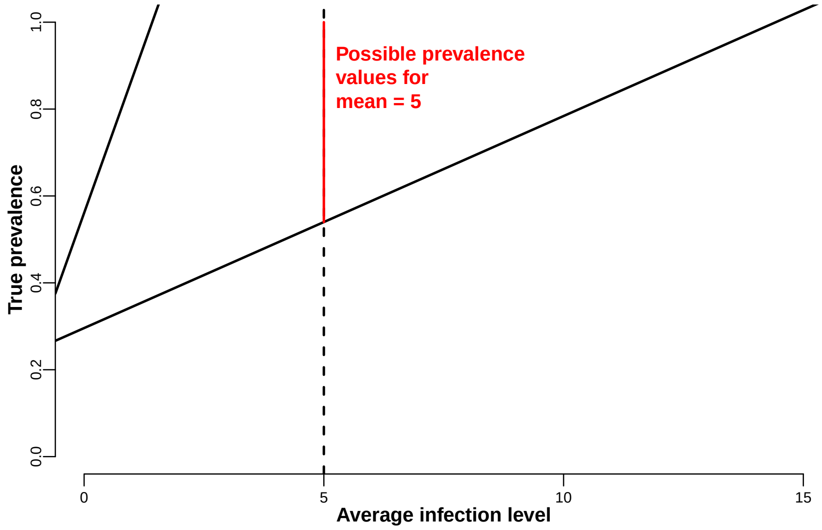

Figure S2.2: Possible values of true prevalence for a given sampled average intensity of infection in the population of 5.
